# The NLRP3 inhibitor NT‐0796 enhances and sustains GLP‐1R agonist‐mediated weight loss in a murine diet‐induced obesity model

**DOI:** 10.1002/oby.24305

**Published:** 2025-04-30

**Authors:** Peter Thornton, Valérie Reader, Zsofia Digby, John Doedens, Nicola Lindsay, Nicholas Clarke, Alan P. Watt

**Affiliations:** ^1^ NodThera Cambridge UK; ^2^ NodThera Seattle Washington USA

## Abstract

**Objective:**

In order to investigate whether a central nervous system penetrant anti‐inflammatory could augment or sustain obesity treatment with semaglutide (Wegovy), a glucagon‐like peptide‐1 receptor (GLP‐1R) agonist, we tested two hypotheses in models of diet‐induced obesity (DIO): 1) a centrally penetrant NLPR3 inhibitor, NT‐0796, drives enhanced weight loss when combined with low‐dose semaglutide, compared to monotherapy; and 2) NT‐0796 monotherapy sustains weight loss induced by semaglutide.

**Methods:**

Mice fed a standard high‐fat or a polyunsaturated fatty acid diet served as models of DIO and were dosed with low‐dose semaglutide, NT‐0796, or combinations. Body weight, food intake, peripheral inflammatory markers, and hypothalamic glial fibrillary acidic protein expression were assessed.

**Results:**

Combined dosing of NT‐0796 with semaglutide drove greater weight loss than either monotherapy alone, and this effect was enhanced in mice consuming the polyunsaturated fatty acid diet. In addition, NT‐0796 sharply limited weight regain following cessation of semaglutide therapy and normalized markers of both peripheral inflammation and hypothalamic astrogliosis to a far greater extent than either semaglutide or calorie restriction.

**Conclusions:**

Alleviation of obesity‐associated inflammation via NLRP3 inhibition 1) constitutes an effective weight‐loss strategy as monotherapy in mice with DIO, 2) augments the weight‐loss efficacy of a subtherapeutic dose of semaglutide, and 3) blocks recovery of lost weight following cessation of semaglutide.

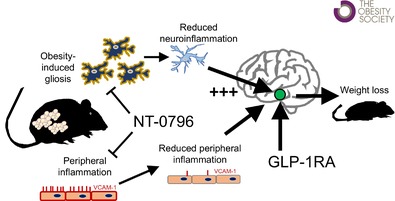

## Graphical abstract

In mouse diet‐induced obesity models, the brain penetrant NLRP3 inhibitor NT‐0796 enhances and sustains the efficacy of the GLP‐1RA semaglutide, leading to a complete reversal of obesity. This correlates with reduced peripheral inflammation and anti‐neuroinflammatory activities, including a reduction in hypothalamic gliosis suggesting further benefits from a NT‐0796/semaglutide combination.

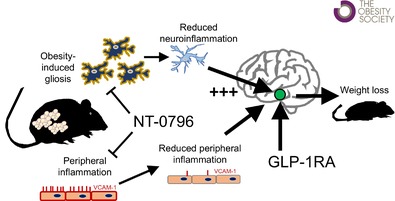

**Study Importance**

**What is already known?**

- Previously, we have demonstrated antiobesity efficacy via NLRP3 inhibition in mouse diet‐induced obesity (DIO) models.
- The antiobesity effect of NLRP3 inhibition was dependent on central nervous system exposure of the two drugs used (i.e., NT‐0796 and NT‐0249).

**What does this study add?**

- In mouse DIO models, the NLRP3 inhibitor NT‐0796 augments the weight‐loss efficacy of semaglutide, and this effect correlates with a reduction in hypothalamic gliosis.
- NT‐0796 ameliorates weight regain following cessation of semaglutide therapy.

**How might these results change the direction of research or the focus of clinical practice?**

- Our clinical‐stage candidate NT‐0796 has the potential to augment existing therapies for obesity and its comorbidities.
- NT‐0796 is uniquely positioned to positively modulate the efficacy of incretin mimetics, with consequent potential to lower their clinical dose and thereby enhance tolerability.

## INTRODUCTION

The prevalence of obesity has nearly tripled in women and quadrupled in men over the past 50 years, and it now affects more than 1 billion people worldwide [1]. The introduction of dual agonist drugs such glucagon‐like peptide‐1 receptor/glucose‐dependent insulinotropic peptide receptor agonists (GLP‐1RAs/GIPR) for the treatment of obesity and type 2 diabetes [2] highlights the impressive efficacy that is now achievable [3]. Although this superior efficacy does not come at the cost of comparably increased gastrointestinal adverse reactions [4], such reactions are still reported [4]. These observations have led to a global search for drug combinations that combine GLP‐1RAs with one or more other drugs that target other pathways with the goal of increasing efficacy with fewer side effects. Examples in development include combinations of GLP‐1RAs with agonists of receptors for amylin, apelin, calcitonin, myostatin, insulin, fibroblast growth factors, and peroxisome proliferator‐activated receptor (PPAR), as well as the melanocortin‐4 receptor and cannabinoid type 1 receptor [5, 6, 7, 8, 9, 10, 11, 12].

In rodent models, high‐fat diet (HFD) feeding drives gliosis in hypothalamic areas involved in energy homeostasis, and this is implicated in obesity pathogenesis [13, 14, 15]. Previous studies have implicated the NOD‐, LRR‐, and pyrin domain‐containing protein 3 (NLRP3) inflammasome in this systemic [16, 17] and hypothalamic inflammation [18]. Critical evidence in support of this hypothesis includes findings stemming from the use of our clinical‐stage NLRP3 inhibitor NT‐0796 (a unique ester prodrug with high tissue and brain penetrance) [19]. This drug exhibits preclinical antiobesity efficacy that associates with reduced hypothalamic gliosis [18]. However, whether NLRP3 inhibition can augment the antiobesity efficacy of GLP‐1RAs, as has recently been claimed [20, 21], remains to be formally investigated.

Discontinuation of antiobesity drugs such as semaglutide often results in the rapid recovery of lost weight [22, 23], and the number of patients who discontinue GLP‐1RA therapy within 2 years is substantial [24]. These observations, combined with the relatively common occurrence of side effects, highlight the need for therapeutic alternatives with the potential to sustain weight loss after GLP‐1RAs have been discontinued.

With this goal in mind, the current study was undertaken to determine whether administration of NT‐0796 can either augment or sustain weight loss induced by a low dose of semaglutide.

## METHODS

### Animals

The efficacy of the NLRP3 inhibitor, NT‐0796, an ester prodrug, requires target cell carboxylesterase (CES) expression for full activity. Therefore, our studies were performed in hCES1 (human carboxylesterase 1) mice, which lack murine plasma *Ces1c* and express human CES1 in myeloid cells [25]. This strain originated and has been maintained at HD Biosciences Co. Ltd. on a C57BL/6 × 129 background as a homozygous colony.

#### Diet‐induced obesity model and compound dosing

The standard chow diet, HFD (60% of kilocalories from fat), and animal group randomizations were as previously described [18]. Additional mouse cohorts were fed a diet enriched in polyunsaturated fatty acids (PUFA; 40% of kilocalories from fat; Dyets, Inc., category #D240510) [26], which bears greater resemblance to the composition of a typical human diet.

#### Statistical analysis

Statistical analysis was performed in GraphPad Prism version 10.2.2 (GraphPad Software) using one‐ or two‐way ANOVA followed by Dunnett or Tukey post hoc or Pearson correlation analysis. A *p* value of ≤0.05 was considered statistically significant. Unless indicated, significance values are for comparison of the indicated condition to diet‐induced obesity (DIO) control mice.

Further methods are detailed in the online Supporting Information.

### RESULTS

#### Effects of the NLRP3 inhibitor, NT‐0796, on efficacy of low‐dose semaglutide in a mouse model of DIO

**FIGURE 1:**
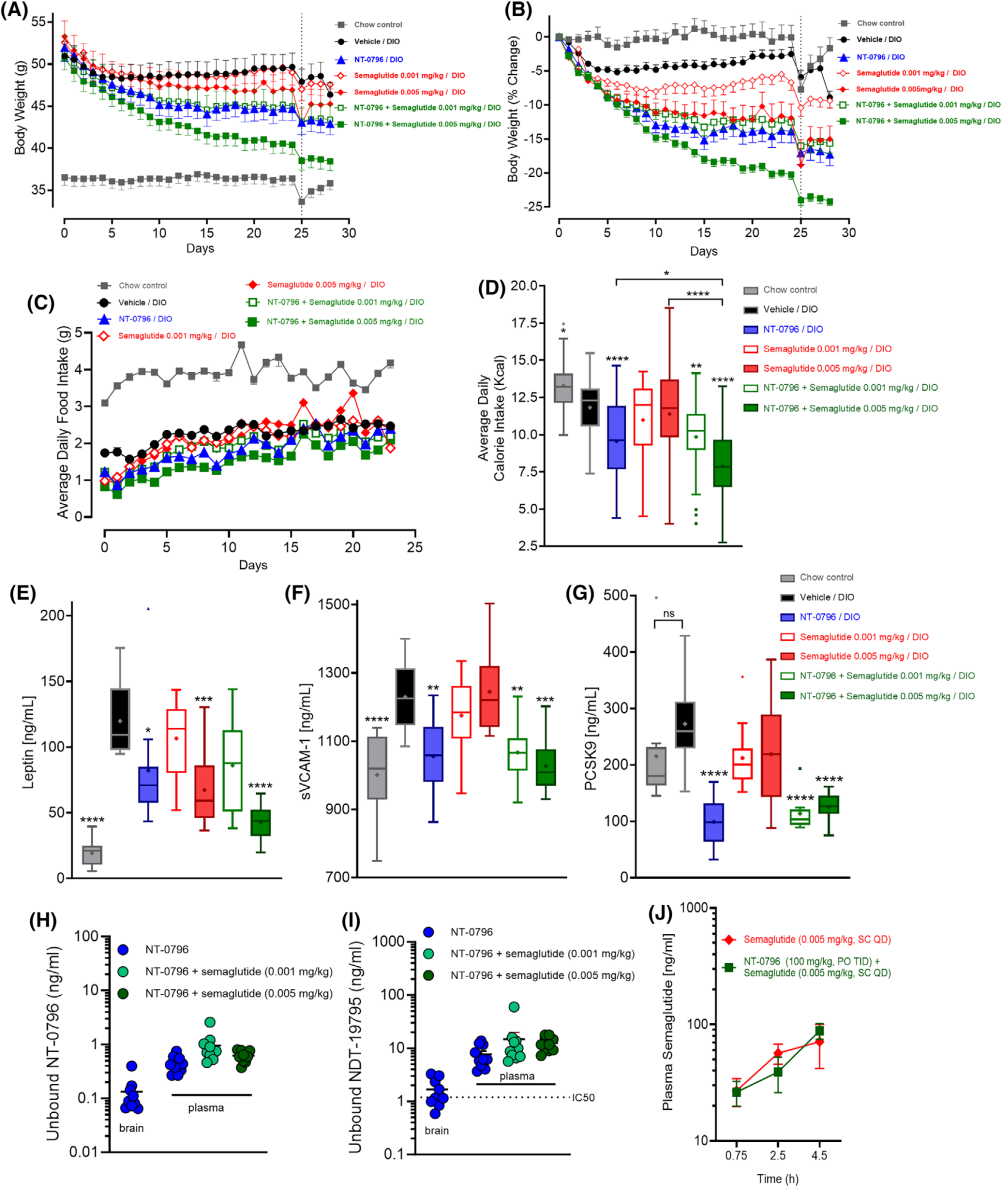
Effects of NT‐0796 or semaglutide as monotherapy or the combination in mice with DIO. Mice with DIO fed a high‐fat diet were dosed therapeutically with NT‐0796 (100 mg/kg, po, tid), semaglutide (0.001 or 0.005 mg/kg, sc, qd), semaglutide (0.001 or 0.005 mg/kg) in combination with NT‐0796, or respective vehicle for 28 days. (A) Absolute body weight. (B) Percentage body weight change from day 0. (C) Average daily food and (D) average daily calorie (kilocalories) intake were calculated across days 0 to 28. (E) Plasma leptin, (F) sVCAM‐1, and (G) PCSK9 were assessed at study end. Unbound brain and plasma concentrations of (H) NT‐0796 and (I) NDT‐19795 in mice with DIO dosed with NT‐0796 with or without semaglutide (0.001 or 0.005 mg/kg). (J) Plasma concentrations of semaglutide in naïve mice dosed to steady state with semaglutide (0.005 mg/kg), with or without NT‐0796. An overnight fast into day 25 (dashed line in panels A and B) was performed for assessment of further exploratory endpoints shown in Figure S1C–F. Data are expressed as mean ± SEM or as box and whisker plots and analyzed by one‐ or two‐way ANOVA with Tukey multiple comparisons test and significance calculated using GraphPad Prism version 10.2.2. ****p < 0.0001; ***p < 0.001; **p < 0.01; and *p < 0.05. DIO, diet‐induced obesity; PCSK9, proprotein convertase subtilisin/kexin type 9; po, orally; qd, 1 time/day; sc, subcutaneously; sVCAM‐1, soluble vascular cell adhesion molecule 1; tid, 3 times/day.

We first tested the weight‐loss efficacy of NT‐0796 alongside two submaximal doses (i.e., 0.001 and 0.005 mg/kg) of semaglutide in mice with DIO. These doses, equivalent to 0.2 and 1.2 nmol/kg/day, respectively, correspond to approximately ED_20_ and ED_50_ (effective doses) of semaglutide [27]. After 15 weeks of HFD feeding, the effects of NT‐0796 (100 mg/kg orally and three times a day) were compared to that of either semaglutide monotherapy or combinations of the two drugs (Figure 1A). Each mouse received both a single daily subcutaneous injection of semaglutide (or corresponding vehicle) and oral gavage of NT‐0796 (or corresponding vehicle) three times daily (Figure S1A). NT‐0796 caused significant weight loss compared to that of mice that were vehicle‐treated (Figure 1A), achieving a maximal 17.3% reduction in body weight by day 28 (Figure 1B and S1B for statistical output). The higher dose of semaglutide (0.005 mg/kg) drove a similar 15.0% reduction of body weight, whereas the lower semaglutide dose (0.001 mg/kg) was largely ineffective compared to that observed in controls and thereby serves as a “subthreshold dose” for the purpose of this study (Figure 1A,B). A key point is that although the combination of NT‐0796 with the subthreshold dose of semaglutide (0.001 mg/kg) was no more effective than NT‐0796 given alone (Figure 1A,B), the combination of NT‐0796 with the higher semaglutide dose (0.005 mg/kg) achieved significantly greater weight loss than either monotherapy alone, detectable as early as day 10 (Figure 1A) and reaching 24.2% weight loss by day 28 (Figure 1B). Thus, semaglutide‐induced weight loss is augmented considerably by NLRP3 inhibition.

Relative to vehicle‐treated mice with DIO, weight loss induced by NT‐0796 was associated with reduced mean daily calorie intake (Figure 1C,D), whereas intake was not significantly reduced by either semaglutide dose over the course of the 28‐day study (Figure 1D). Just as weight loss was augmented by combining the NLRP3 inhibitor with the higher semaglutide dose, so too was the reduction of calorie intake compared to either monotherapy alone (Figure 1D). In contrast, the effect of combination therapy to enhance calorie intake suppression was not observed among mice receiving NT‐0796 and the lower semaglutide dose (Figure 1D). These data implicate reduced food intake in the mechanism underlying NT‐0796‐induced weight loss, whether given by itself or in combination with semaglutide. Importantly, we have previously shown that these effects of NT‐0796 on food intake and body weight loss are not observed in normal chow‐fed control mice [18], thereby supporting the hypothesis that obesity‐associated NLRP3 inflammation is required for NT‐0796 efficacy.

Plasma leptin levels were elevated in mice with DIO relative to chow‐fed controls, and, as expected, this increase was reduced among all groups of mice that experienced significant weight loss. Thus, mean plasma leptin levels on day 28 were reduced by 37.4% or 57.3% following treatment with NT‐0796 or semaglutide (0.005 mg/kg), respectively (Figure 1E), with an even greater effect (i.e., 76.6% lower levels) observed in mice in which NT‐0796 and the higher dose of semaglutide (0.005 mg/kg) were combined (Figure 1E). In contrast, plasma leptin levels were not significantly reduced in mice receiving the lower‐dose semaglutide, whether given alone or in combination with NT‐0796 (Figure 1E).

In order to confirm NT‐0796 target engagement, we measured the previously characterized NLRP3‐sensitive biomarkers, i.e., the plasma levels of proprotein convertase subtilisin/kexin type 9 (PCSK9) and soluble vascular cell adhesion p 1 (sVCAM‐1; a marker of peripheral vascular inflammation) [18]. As expected, plasma levels of sVCAM‐1 were elevated in mice with DIO compared to chow‐fed controls, and NT‐0796 monotherapy drove reductions of this plasma marker of 76.1% when given alone and either 71.4% or 88.6% when given in combination with either the 0.001‐ or 0.005‐mg/kg semaglutide dose, respectively (Figure 1F). In contrast, semaglutide monotherapy was largely ineffective at lowering sVCAM‐1 (Figure 1F). Plasma PCSK9 levels were also unaffected by semaglutide in mice with DIO but strongly inhibited by NT‐0796 whether given as monotherapy or in combination with either dose of semaglutide (Figure 1G). These data 1) confirm the efficacy of NT‐0796 as an NLRP3 inflammasome inhibitor, 2) suggest that DIO activates the NLRP3 inflammasome, resulting in elevated plasma levels of sVCAM‐1 and PCSK9, and 3) show that, despite its weight‐loss efficacy, semaglutide has little impact on this inflammatory response to HFD consumption.

As the effect of semaglutide to inhibit gastric emptying could potentially limit oral bioavailability/exposure of NT‐0796, we compared terminal blood levels of NT‐0796 (and its active metabolite, NDT‐19795) [19] between animals that did and did not receive semaglutide. Following NT‐0796 dosing, free levels of NT‐0796 (Figure 1H) and NDT‐19795 (Figure 1I) were detectable in both brain and plasma, with NDT‐19795 achieving concentrations above its in vivo half‐maximal inhibitory concentration (IC_50_) [18] in both compartments. Furthermore, these levels were not significantly impacted by semaglutide coadministration at either dose (Figure 1H,I). We also evaluated semaglutide exposures in naïve mice (Figure 1J), dosed to steady state for 3 days with semaglutide (0.005 mg/kg) or in combination with NT‐0796. Semaglutide pharmacokinetics were unaffected by coadministration of NT‐0796 (Figure 1J).

Relative to vehicle‐treated mice with DIO, NT‐0796 also drove improvements in glucose homeostasis and insulin sensitivity, as measured following either an oral glucose tolerance test (Figure S1C,D) or insulin tolerance test (Figure S1E,F). Semaglutide‐dosed mice, at 0.001 or 0.005 mg/kg, also displayed significant improvements in both glucose homeostasis and insulin resistance, and there were no apparent additive or synergistic effects of NT‐0796 on these parameters when administered with semaglutide (Figure S1C–F). Although additional investigation is needed, we infer that these metabolic benefits arise primarily from drug‐induced weight loss (rather than from a weight‐independent action of the drug).

#### Weight regain following curtailment of semaglutide dosing

**FIGURE 2:**
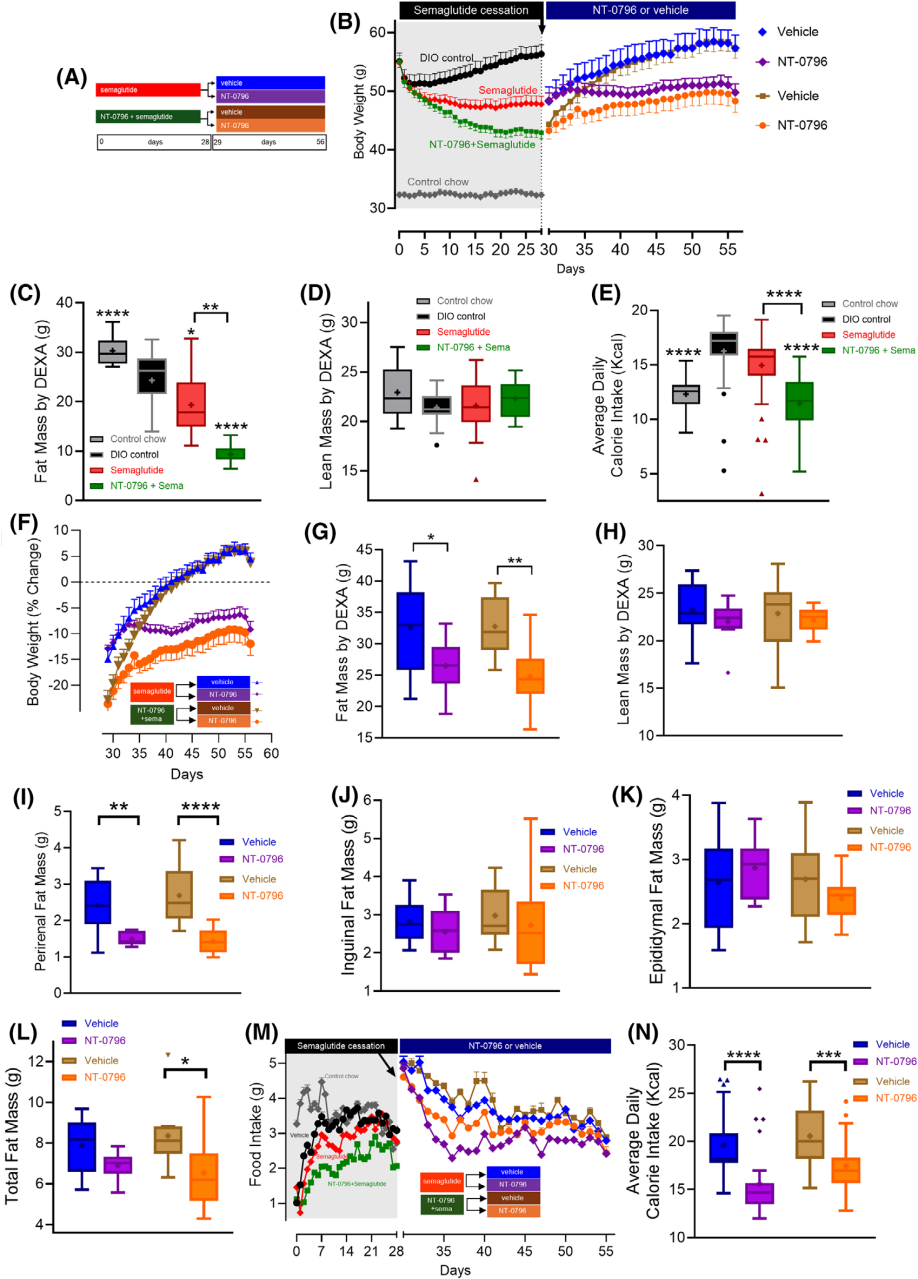
Effects of switching from semaglutide to NT‐0796 therapy in mice with DIO. Mice with DIO fed a high‐fat diet were dosed therapeutically with semaglutide (0.005 mg/kg, sc, qd) or a combination of NT‐0796 (100 mg/kg, po, tid) and semaglutide (0.005 mg/kg, sc, qd) for 28 days. Following 28 days, one‐half of each group was reassigned to receive NT‐0796 (100 mg/kg, po, tid) or vehicle (po, tid) alone until study end (day 56). (A) Experimental schematic. (B) Body weight following semaglutide or the NT‐0796 and semaglutide combination (days 0–28) and following the switch to NT‐0796 or vehicle (29–56 days). (C) Fat mass and (D) lean mass were evaluated by body composition DXA scans at day 28. (E) Average daily calorie intake (kilocalories; 0–28 days). (F) Percentage body weight change (29–56 days). (G) Fat mass and (H) lean mass were evaluated by body composition DXA scans at day 56. (I) Perirenal, (J) inguinal, (K) epididymal, and (L) total fat mass was quantified at study end (day 56). (M) Average daily food intake was assessed over days 0 to 56. (N) Average daily calorie intake (kilocalories) was assessed in the period following semaglutide cessation (days 29–56). Data are expressed as mean ± SEM or as box and whisker plots and analyzed by one‐way ANOVA with Tukey multiple comparisons test and significance calculated using GraphPad Prism version 10.2.2.****p < 0.0001; ***p < 0.001; **p < 0.01; and *p < 0.05. DEXA, dual‐energy x‐ray absorptiometry; DIO, diet‐induced obesity; po, orally; qd, 1 time/day; sc, subcutaneously; tid, 3 times/day.

We next determined whether NT‐0796 could prevent weight regain following cessation of either semaglutide or NT‐0796/semaglutide combination dosing (Figure 2A and Figure S2A). As before, weight loss was greater when semaglutide (0.005 mg/kg) was combined with NT‐0796 than that in mice receiving semaglutide (0.005 mg/kg) monotherapy (22.0% vs. 13.1%; *p* < 0.05, Figure 2B and Figure S2B for statistical output). Dual‐energy X‐ray absorptiometry (DXA) scans on day 28 confirmed that, compared to semaglutide monotherapy, fat mass loss was increased in mice on the combination dosing regimen (Figure 2C). In contrast, lean mass was preserved in response to treatment (Figure 2D). Calorie intake was also significantly reduced in combination‐dosed mice relative to semaglutide monotherapy (Figure 2E). We then assessed weight regain following cessation of semaglutide in both groups on day 29, with mice receiving either NT‐0796 or its vehicle for a further 28 days (Figure 2A and detailed information in Figure S2A).

As expected, the switch from combination therapy to vehicle alone (beginning on day 29) resulted in a body weight increase from 22.8% below the baseline value (on day 29) to 3.6% over baseline by study end (Figure 2B,F). By comparison, the degree of weight regain following cessation of combination therapy was reduced by ~50% (to a value 11.9% below baseline body weight) in mice maintained on the same dose of NT‐0796 as monotherapy (Figure 2F). DXA scans performed at study end (day 56) confirmed that maintenance of NT‐0796 dosing limited the associated increase of fat mass (Figure 2G), while preserving lean mass (Figure 2H), relative to vehicle‐treated controls. A similar pattern was observed in mice with DIO following the switch from semaglutide monotherapy to vehicle, with body weight (Figure 2F) increasing from 14.9% below their baseline value (day 29) to 4.4% over baseline by study end (Figure 2F). Again, this recovery of lost weight following cessation of semaglutide monotherapy was limited following the switch to NT‐0796 administration, with mice remaining 9.2% below their baseline body weight at study end, an ~70% decrease in the amount of weight that was regained (Figure 2F). Consistent with this effect, reductions of absolute fat mass (perirenal fat mass, Figure 2I; inguinal fat mass, Figure 2J; epididymal fat mass, Figure 2K; total fat mass, Figure 2L) were also observed in mice with DIO that switched from semaglutide (monotherapy or combination) to NT‐0796 relative to vehicle‐treated controls. Liver, heart, and kidney (but not brain) mass also trended lower in mice maintained on NT‐0796 relative to vehicle‐treated controls (Figures S2C–G). Despite an initial rise in all groups following cessation of semaglutide (as monotherapy or combination therapy), mean daily calorie intake was significantly reduced in mice maintained on NT‐0796 relative to vehicle (Figure 2M,N). Finally, fasting levels of blood glucose, insulin, glucagon, and homeostatic model assessment of insulin resistance (HOMA‐IR) were reduced in mice maintained on NT‐0796 following semaglutide cessation compared to vehicle‐treated controls (Figure S2H–K).

**FIGURE 3:**
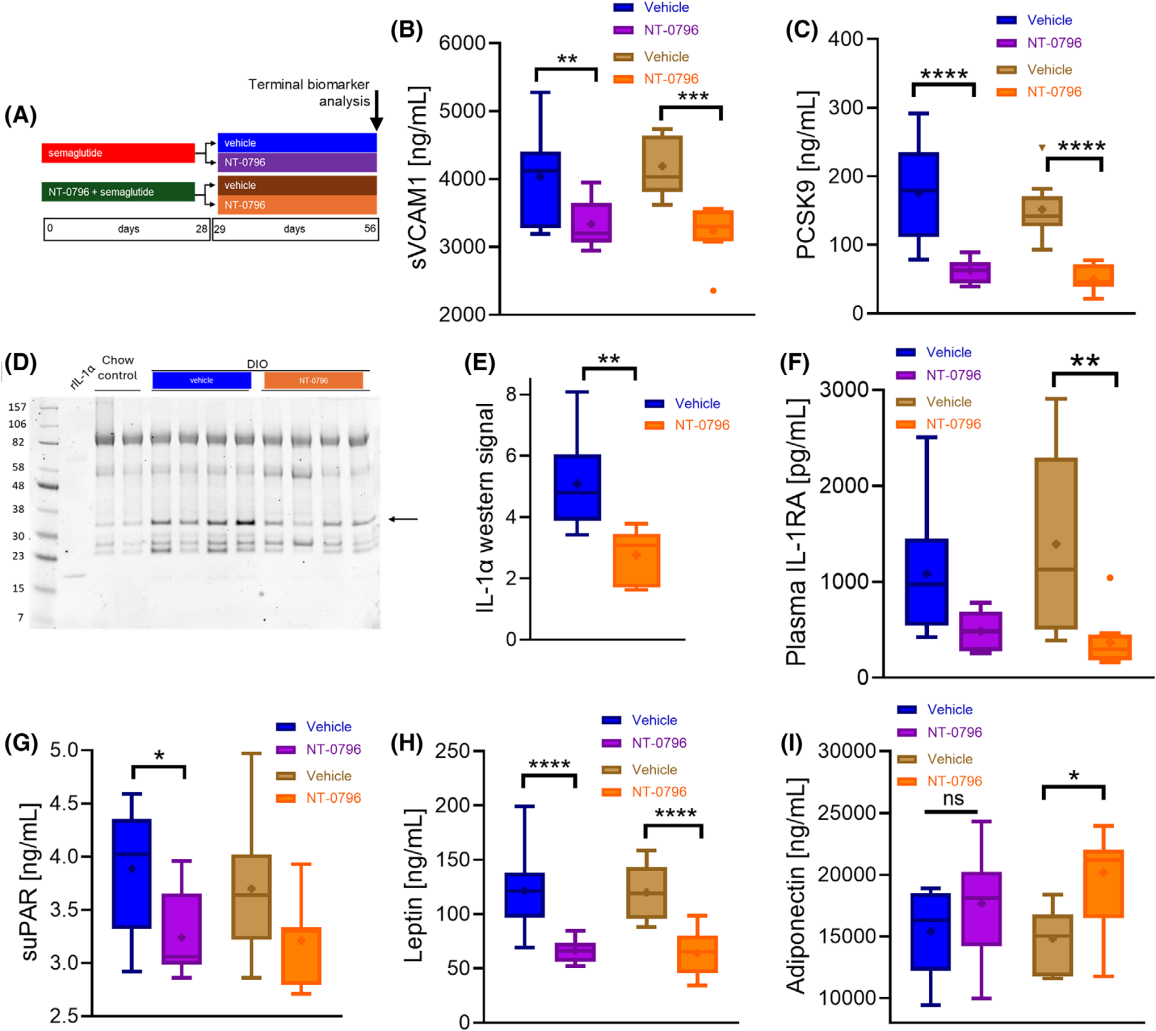
Effects of switching from semaglutide to NT‐0796 on systemic, hepatic, and cardiovascular inflammatory markers following cessation of semaglutide in mice with DIO. Mice with DIO were maintained on NT‐0796 (100 mg/kg, po, tid) or vehicle treatment for 28 days following an initial period of semaglutide or NT‐0796/semaglutide therapy. (A) Experimental schematic: terminal samples were analyzed for biomarkers of inflammation (B) sVCAM‐1 and (C) PCSK9. (D) Liver IL‐1α expression was assessed by Western blotting (arrow corresponds to 31‐kDa full‐length protein). (E) Densitometric assessment of full‐length liver IL‐1α from Western blot images. Plasma levels of (F) IL‐1RA, (G) suPAR, (H) leptin, and (I) adiponectin were assessed. Data are expressed as box and whisker plots and analyzed by one‐way ANOVA with Tukey multiple comparisons test and significance calculated using GraphPad Prism version 10.2.2. ****p < 0.0001; ***p < 0.001; **p < 0.01; and *p < 0.05. DIO, diet‐induced obesity; IL‐1RA, interleukin‐1 receptor antagonist; PCSK9, proprotein convertase subtilisin/kexin type 9; po, orally; suPAR, urokinase plasminogen activator surface receptor; sVCAM‐1, soluble vascular cell adhesion molecule 1; tid, 3 times/day.

Peripheral NLRP3‐dependent inflammatory markers sVCAM‐1 (Figure 3B) and PCSK9 (Figure 3C) were reduced by continuing NT‐0796 dosing relative to vehicle‐treated controls. Previously, we were unable to detect robust induction of NLRP3‐derived interleukin (IL)‐1β or downstream IL‐6 in blood of mice with DIO [18]. However, to further characterize target engagement by NT‐0796 within tissues, we explored the expression of IL‐1 cytokines in livers of mice with DIO. Despite being unable to detect IL‐1β within liver tissues (not shown), we detected liver IL‐1α, the hepatic expression of which is under the control of NLRP3‐dependent cytokine IL‐1β (Figure 3D) [28]. Mice remaining on NT‐0796 monotherapy maintained reduced liver IL‐1α levels relative to vehicle‐treated controls (Figure 3D,E). In support of this, the acute phase protein IL‐1RA, a liver‐derived marker that we have previously characterized as an NLRP3 activation marker in obesity models [18], was also reduced by NT‐0796 (Figure 3F). Furthermore, the cardiovascular marker urokinase plasminogen activator surface receptor (suPAR; Figure 3G) and leptin (Figure 3H) were similarly reduced among mice switched to NT‐0796 therapy (relative to vehicle), whereas plasma adiponectin levels tended to increase (Figure 3I). Together, these data show that the beneficial effects of semaglutide in mice with DIO are largely preserved by switching to NT‐0796 dosing and that NLRP3 inhibition provides marked reductions in biomarkers related to vascular inflammation and acute phase response.

#### Combined effects of NT‐0796 and semaglutide in mice with DIO during exposure to a PUFA diet

**FIGURE 4:**
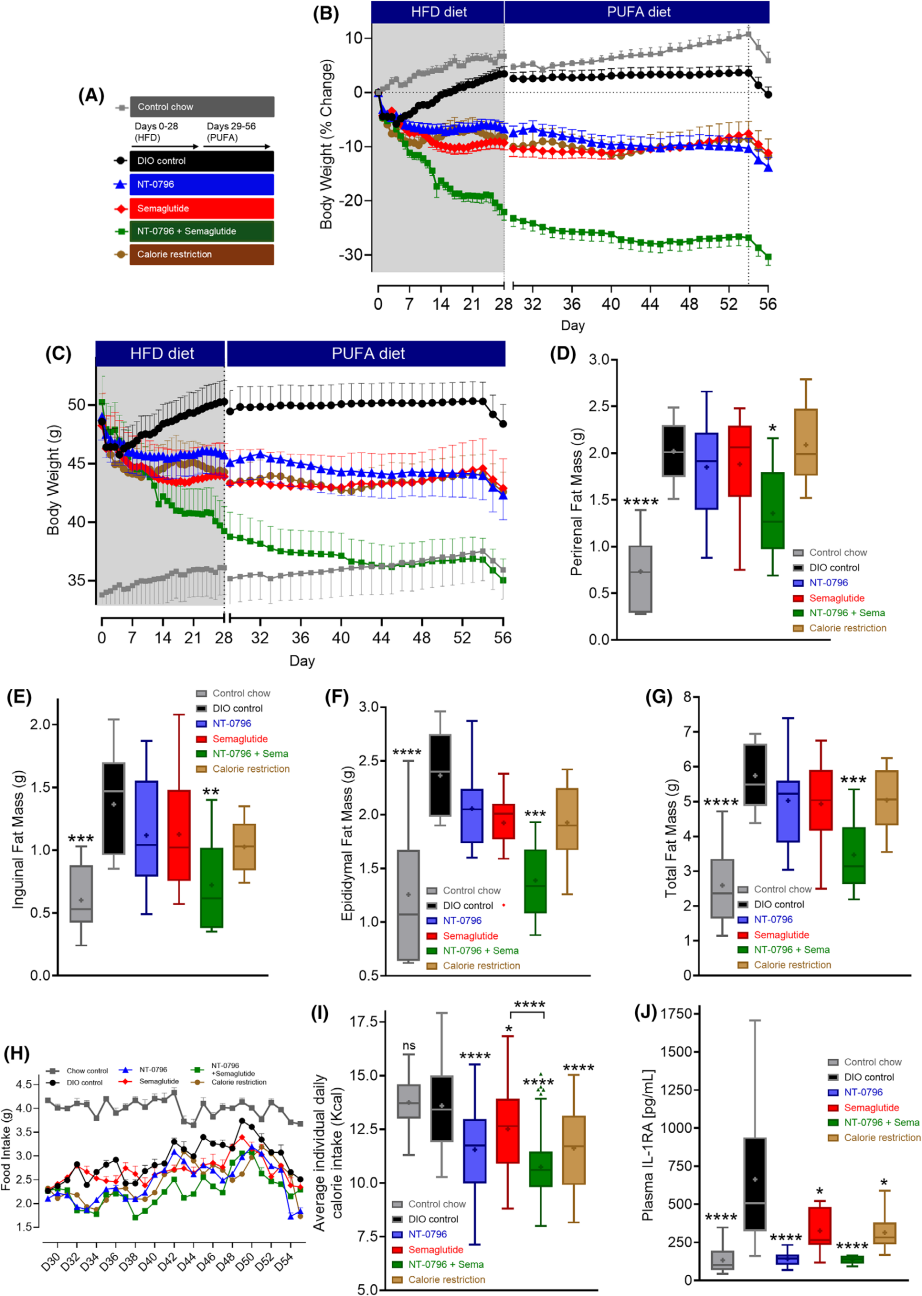
Effects of NT‐0796, semaglutide, or combinations in mice with DIO switched to a PUFA diet. Mice with DIO fed a HFD were dosed therapeutically with NT‐0796 (100 mg/kg, po, tid), semaglutide (0.005 mg/kg, sc, q.d), or a combination from day 0 to 28. On day 29, diet was switched to a PUFA diet, and the respective treatment continued (over days 29–56). An additional group of mice served as calorie‐restricted controls, body weight of which were maintained as closely to NT‐0796‐dosed mice as possible by controlling the degree of calorie restriction throughout the experiment. (A) Experimental schematic. (B) Percentage body weight change over time. An overnight fast into day 55 (dashed line) was performed for assessment of further exploratory endpoints. (C) Body weight over time. (D) Perirenal, (E) inguinal, (F) epididymal, and (G) total fat mass was assessed at study end (day 56). (H) Average daily food intake and (I) average daily calorie (kilocalories) intake during PUFA diet exposure (days 29–56). (J) Plasma IL‐1RA levels at study end (day 56). Data are expressed as mean ± SEM or as box and whisker plots and analyzed by one‐ or two‐way ANOVA with Tukey multiple comparisons test and significance calculated using GraphPad Prism version 10.2.2. ****p < 0.0001; ***p < 0.001; **p < 0.01; and *p < 0.05. DIO, diet‐induced obesity; HFD, high‐fat diet; IL‐1RA, interleukin‐1 receptor antagonist; po, orally; PUFA, polyunsaturated fatty acid; qd, 1 time/day; sc, subcutaneously; tid, 3 times/day.

In order to assess the efficacy of NT‐0796/semaglutide combination therapy in a more translationally relevant DIO model, a subset of the studies described earlier was repeated in mice fed a less‐inflammatory PUFA‐enriched diet (40% fat vs. HFD, which included 60% fat). After repeating the 28‐day protocol shown in Figure 1, HFD‐fed mice were switched onto the PUFA diet for a further 28 days (Figure 4A and Figure S3A). This approach enabled us to determine the weight‐loss efficacy of the drug interventions across the two diets but within the same cohort of mice. Mice were treated with NT‐0796 alone, semaglutide (0.005 mg/kg) alone, or a combination of the two. We also included a weight‐matched control group that was fed the same PUFA diet but was calorie‐restricted to match the body weight of NT‐0796–treated mice.

As was observed in mice fed the HFD (Figure 1), NT‐0796 and semaglutide (0.005 mg/kg) each drove significant weight loss, reaching −6.7% and −9.3% of baseline weight, respectively, by day 28 (Figure 4B and Figure S3B for statistical output). By comparison, the 21% weight loss induced by the NT‐0796/semaglutide combination was more than double that achieved by either monotherapy alone (Figure 4B,C and Figure S3B for statistical output). This was confirmed by DXA scanning, which showed that percent body fat was most significantly reduced (relative to DIO controls at day 28) in mice on combination therapy (Figure S3C). Lean mass was preserved (relative to DIO controls) across drug intervention groups, despite the changes in fat mass modulation (Figure S3D). Interestingly, combination therapy also yielded the greatest preservation of bone mineral content relative to DIO controls (Figure S3E).

After switching onto the PUFA diet on day 29, formerly HFD‐fed controls maintained their weight, as did mice that remained on semaglutide monotherapy. In contrast, mice maintained on NT‐0796 continued to lose weight after the switch from the HFD to the PUFA diet, such that, by day 56, they weighed 13.8% less than their baseline body weight (Figure 4B). By design, the calorie‐restricted group achieved a level of weight loss comparable with the NT‐0796 dosed group, amounting to an 11.8% reduction by day 56 (Figure 4B).

When mice that were maintained on combined dosing of NT‐0796 and semaglutide were switched onto the PUFA diet, weight loss continued such that, by day 43, the obesogenic state was completely reversed, and mice weighed the same as chow‐fed controls (corresponding to a 30.3% weight loss; Figure 4B,C and Figure S3B for statistical output). This effect was once again more than double that achieved by either monotherapy alone. Although calorie intake was reduced in all drug‐treated groups among mice fed the PUFA diet, this effect was also greater among mice receiving the NT‐0796/semaglutide combination (Figure 4H,I). Similarly, perirenal (Figure 4D), inguinal (Figure 4E), epididymal (Figure 4F), and total (Figure 4G) fat mass was each reduced to a greater extent by the NT‐0796/semaglutide combination than by either drug alone.

Regarding markers of peripheral inflammation, NT‐0796 either as monotherapy or in combination with semaglutide was once again highly effective in reducing obesity‐driven increases in plasma IL‐1RA (a marker of peripheral NLRP3 activity; Figure 4J). By comparison, weight loss induced by semaglutide monotherapy was associated with a more modest reduction of plasma IL‐1RA levels (Figure 4J), an effect comparable with that of calorie restriction (Figure 4J). HOMA‐IR at study end was most consistently reduced by NT‐0796 monotherapy, or NT‐0796 in combination with semaglutide, but these did not reach statistical significance (Figure S3F).

#### Assessment of hypothalamic gliosis

**FIGURE 5:**
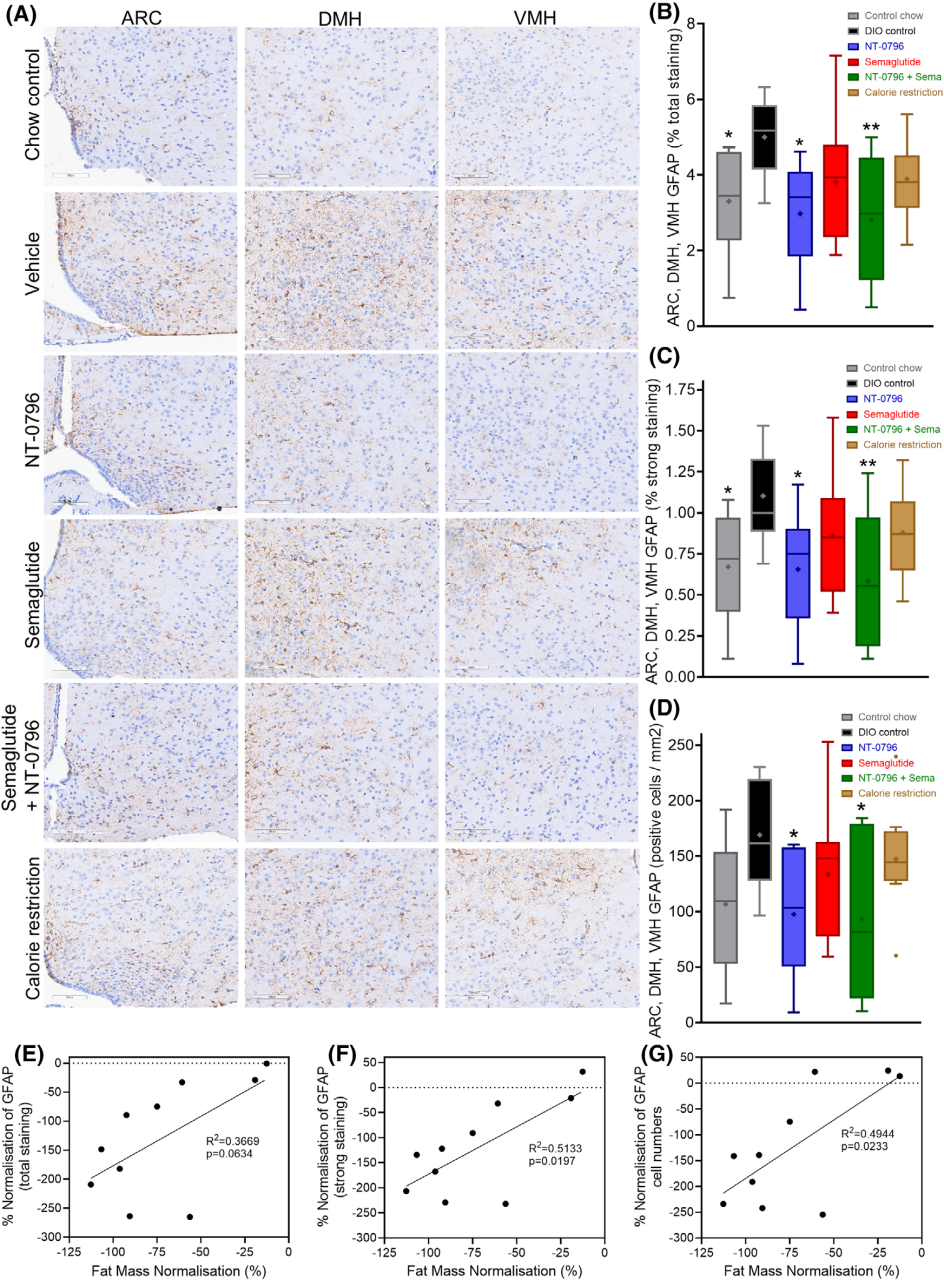
Effects of NT‐0796, semaglutide, or a combination on hypothalamic GFAP. (A) GFAP immunoreactivity was assessed within the ARC, DMH, and VMH of mice with DIO following continuous dosing with NT‐0796 (100 mg/kg, po, tid), semaglutide (0.005 mg/kg, sc, qd), their combination, or calorie restriction over 56 days in mice with DIO. Representative images taken from within the regions of interest are shown. (B) Average total GFAP staining (percent), (C) average strong GFAP staining (percent), or (D) average total GFAP‐positive cell numbers (per millimeters squared) across the ARC, DMH, and VMH. Pearson correlation analysis among the percent normalization of total fat mass and percent normalization of (E) total GFAP staining, (F) strong GFAP staining, or (G) GFAP cell numbers (across the ARC, DMH, and VMH) following the combined dosing of NT‐0796 and semaglutide. Data are expressed as box and whisker plots and analyzed by one‐way ANOVA with Tukey multiple comparisons test and significance calculated using GraphPad Prism version 10.2.2. **p < 0.01; and *p < 0.05. ARC, arcuate nucleus; DIO, diet‐induced obesity; DMH, dorsomedial hypothalamus; GFAP, glial fibrillary acidic protein; HFD, high‐fat diet; po, orally; qd, 1 time/day; sc, subcutaneously; tid, 3 times/day; VMH, ventromedial hypothalamus.

We have previously reported that, in mice with DIO, brain penetrance and anti‐neuroinflammatory actions of NT‐0796 are tied to its weight‐loss efficacy [18]. Dysregulated signaling within the mediobasal hypothalamus (MBH) can contribute to obesity pathogenesis, and clinical imaging studies of participants with obesity support the coexistence of gliosis within this region [29, 30]. We reasoned that inhibition of a collective astrocytic activation score (pertaining to the arcuate nucleus [ARC], dorsomedial hypothalamus [DMH], and ventromedial hypothalamus [VMH]) by NT‐0796 would be consistent with its reduction of aberrant glial signaling within the MBH. Therefore, we quantified immunohistochemical staining of glial fibrillary acidic protein (GFAP) across three hypothalamic areas (i.e., ARC, DMH, and VMH) at the conclusion of the PUFA diet study. Relative to chow‐fed controls, mean values of total percent GFAP staining, percentage of strongly stained GFAP, and total GFAP‐positive cell numbers were elevated in control mice with DIO (Figure 5A–D). This gliosis reaction was significantly blunted across all three hypothalamic regions by treatment with NT‐0796, whether as monotherapy or in combination with semaglutide (Figure 5B–D). Calorie restriction or semaglutide monotherapy also tended to reduce total GFAP staining across these regions, but the effect was less than that induced by NT‐0796 and did not achieve statistical significance (Figure 5B–D). Although the decrease of GFAP immunoreactivity specifically in the ARC induced by either semaglutide or calorie restriction did achieve statistical significance (relative to DIO controls; Figure S4D), GFAP staining was reduced more consistently across all hypothalamic areas tested in mice receiving NT‐0796 (Figure S4). Pearson correlation analysis demonstrated that, in mice receiving the NT‐0796/semaglutide combination, GFAP immunoreactivity was fully normalized (as percent total, percent strong, or total cell numbers) in a manner that correlated strongly with reductions in fat mass (Figure 5E–G). Correlations between changes in GFAP immunoreactivity and fat mass for other dose groups did not achieve statistical significance (not shown).

## DISCUSSION

Despite their impressive efficacy, the utility of GLP‐1RA drugs for the treatment of obesity remains limited by issues related to tolerability, availability, and affordability, as well as rapid recovery of lost weight once the drug is discontinued. These considerations highlight the need for therapeutics that engage alternative mechanisms that might be used instead of or in combination with lower doses of GLP‐1RAs.

Across our three HFD studies, low‐dose semaglutide (0.005 mg/kg) drove weight losses of 15.0%, 13.1%, and 9.3% (mean, 12.5%). By comparison, weight loss induced by the combination of semaglutide and NT‐0796 was increased nearly twofold (24.2%, 22.0%, and 22.1%; mean,  22.8%). The extent of benefit conferred by this drug combination appears to be diet‐dependent, in that it was enhanced when the same cohort of mice was switched onto a more translationally relevant PUFA diet (achieving weight loss of 30.3%). Furthermore, the obesogenic state of PUFA‐fed mice with DIO was completely reversed by the combination of NT‐0796 and low‐dose semaglutide. In summary, NT‐0796 augments the weight‐loss efficacy of low‐dose semaglutide monotherapy by 83% (on HFD) and up to 173% (on PUFA diet). For context, we note that, in recent press releases, other NLRP3 inhibitors have been suggested to augment semaglutide‐driven weight loss by ≤50% [20, 21]. These observations highlight the therapeutic benefit conferred by a weight‐loss strategy that combines NLRP3 inhibition with GLP‐1RAs.

PUFA‐enriched diets differ from a standard HFD in several ways. They are less obesogenic in humans [31, 32] and may protect from ectopic lipid storage in rodents [26, 33, 34]. Although beyond the scope of the current manuscript, we speculate that a reduced inflammatory response to the PUFA diet (relative to HFD) lowers obesogenic drive and thereby enhances normalization of aberrant inflammation of NT‐0796. Consistent with this hypothesis, the percentage of strong hypothalamic GFAP immunostaining in response to the PUFA diet was lower compared to our previous data following the HFD [18]. These interpretations also align with the fact that the PUFA diet curtailed the upward trajectory in weight gain in mice previously fed the HFD.

Although mechanisms underlying the weight‐loss benefit of the NT‐0796/semaglutide combination remain to be determined, we interpret our findings to suggest that reductions in neuroinflammation (e.g., GFAP) via NLRP3 inhibition play a key role. This inference is based on the following two observations: 1) the percentage normalization of fat tissue mass induced by dosing of NT‐0796 and semaglutide was strongly correlated with its reduction in GFAP immunoreactivity across the ARC, DMH, and VMH; and 2) in mice receiving either semaglutide or NT‐0796 monotherapy, significant reduction of overall hypothalamic gliosis was detectable only with the latter, even when the degree of weight loss was not substantially different. Together, these findings suggest that at least a component of the reduced gliosis resulting from NLRP3 inhibition was mediated via mechanisms independent of weight loss per se. This is further supported by recent evidence demonstrating that astrocytic deletion of IL‐1 (NLRP3‐dependent cytokine) receptor signaling adaptor MyD88 (myeloid differentiation primary response protein) inhibits obesity pathogenesis in mice [15].

Gliotic responses within the brain, particularly subnuclei within the hypothalamus, have gained substantial attention for their role in obesity [13, 14, 15, 29, 30]. Although incretin mimetics possess anti‐inflammatory attributes, their accessibility to deeper central nervous system structures, besides the arcuate nucleus, is questionable [27, 35]. We believe that the higher brain penetrance of NT‐0796 contributes to its ability to ameliorate gliosis across the MBH, including deeper nuclei such as the DMH and VMH. Because gliosis contributes to obesity pathogenesis [36, 37], it is not surprising that this beneficial effect of NLRP3 inhibition augments the efficacy of GLP‐1RAs, especially because gliosis does not appear to be a key target for the action of the latter drug class. We also note the potential of comorbidities, including diabetes, to exacerbate neuroinflammatory responses within deeper hypothalamic nuclei, as was recently demonstrated in the paraventricular nucleus of diabetic nonhuman primates [38]. This type of progression may contribute to the reduced weight‐loss efficacy of GLP‐1RAs in individuals with type 2 diabetes mellitus [39]. We also found that reductions in HOMA‐IR were most consistently observed in mice dosed with NT‐0796 (as monotherapy or in combination with semaglutide). Taken together, these observations highlight the so far untapped potential of glial‐targeted anti‐inflammatory therapy to increase weight‐loss efficacy.

We also acknowledge evidence that anti‐inflammatory agents such as etanercept can augment GLP‐1R signaling [40]. Thus, it is possible that NT‐0796 enhances semaglutide potency by augmenting GLP‐1R‐mediated signal transduction. Based on these collective considerations, we anticipate that the greater weight‐loss efficacy of combined NLRP3 inhibition/GLP‐1R agonism involves complex neuronal‐glial interactions, and future studies are warranted to identify them.

As previously reported [18], both NT‐0796 and semaglutide elicit weight loss through appetite suppression and reduced calorie intake. However, the disproportionately greater loss of fat mass on combination dosing relative to food intake reduction (Figure 4D–G) suggests additional mechanisms may contribute. One possibility is enhanced energy expenditure or fat oxidation rates, as has been described in caspase‐1^−/−^ mice (a downstream target of NLRP3) [17, 41], and potentially mediated via sympathetic activation [42]. Further studies will be required to fully understand these mechanism(s).

In conclusion, our data support a model wherein ongoing inflammation (including hypothalamic gliosis) represents a druggable mechanism implicated in obesity pathogenesis, one that can be ameliorated by NLRP3 inhibition. We further suggest that such an effect has untapped potential to augment existing therapies for obesity and its comorbidities. Thus, NT‐0796 is uniquely positioned to positively modulate the efficacy of incretin mimetics, with consequent potential to lower their clinical dose and thereby enhance tolerability. We also demonstrate the potential of NT‐0796 as an oral follow‐on medication, reducing markers of vascular and hepatic inflammation, hyperleptinemia, and weight regain in individuals switching away from incretin mimetic therapy.

## FUNDING INFORMATION

NodThera is privately funded with venture capital money. No other external funding has been used.

## CONFLICT OF INTEREST STATEMENT

Peter Thornton, Valérie Reader, Zsofia Digby, Nicola Lindsay, and Alan P. Watt are current employees of NodThera. All authors are equity holders of NodThera. Alan P. Watt is Chair of Babraham Institute Enterprise Board. Alan P. Watt and Valérie Reader have patents for NT‐0796.

## Supporting information


**Data S1.** Supporting Information.

## Data Availability

The data that support the findings of this study are available from the corresponding author upon reasonable request.
