# Supplementary material for: The NLRP3 inhibitor NT‐0796 enhances and sustains GLP‐1R agonist‐mediated weight loss in a murine diet‐induced obesity model
Source: Obesity (Silver Spring). 2025 Apr 30;33(7):1309–21. doi: 10.1002/oby.24305 (PMC12210099; doi:10.1002/oby.24305)
Supplement: Supplementary file 1 — Data S1. Supporting Information. [file OBY-33-1309-s001.pdf]

Supplementary Information

The NLRP3 inhibitor NT-0796 enhances and sustains GLP-1R agonist mediated weight loss in a murine diet-induced obesity model

Peter Thornton, Valérie Reader, Zsofia Digby, John Doedens, Nicola Lindsay, Nicholas Clarke, Alan P. Watt

Affiliations:

PT, ZD, VR, NL, NC, APW**:** NodThera, Suite 8, The Mansion, Chesterford Research Park, Cambridge, CB10 1XL, UK

JD**:** NodThera, 454 N 34th St, Seattle, WA 98103, USA

A)

Group	N	Diet	Compound Treatment 1	Compound Treatment 2	Treatment Duration
Chow control	10	Chow control	NA	NA	NA
Vehicle / DIO	10	HFD (ad libitum)	Vehicle (s.c., q.d.)	Vehicle (p.o., t.i.d; 0-7-14h)	Day 0 to Day 28
NT-0796 / DIO	10	HFD (ad libitum)	Vehicle (s.c., q.d)	NT-0796 (100mpk, p.o., t.i.d.)	Day 0 to Day 28
Sema 0.001 / DIO	10	HFD (ad libitum)	Semaglutide (0.001mpk s.c, q.d.)	Vehicle (p.o., t.i.d.)	Day 0 to Day 28
Sema 0.005 / DIO	10	HFD (ad libitum)	Semaglutide (0.005mpk s.c., q.d.)	Vehicle (p.o., t.i.d.)	Day 0 to Day 28
NT-0796 + Sema (0.001) / DIO	10	HFD (ad libitum)	Semaglutide (0.001mpk s.c., q.d.)	NT-0796 (100mpk, p.o., t.i.d.)	Day 0 to Day 28
NT-0796 + Sema (0.005) / DIO	10	HFD (ad libitum)	Semaglutide (0.005mpk s.c., q.d.)	NT-0796 (100mpk, p.o., t.i.d.)	Day 0 to Day 28

B)

Figure	Comparison		* p<0.05	** p<0.01	*** p<0.001	**** p<0.0001
1A	Vehicle / DIO	Chow control	-	-	-	Day 0-28
	Vehicle / DIO	NT-0796 / DIO	Day 28	-	-	-
	Vehicle / DIO	NT-0796 + Semaglutide 0.005 mg/kg / DIO	Day 10-13	Day14-18	Day 19-21, 28	Day 22-27
	NT-0796 + Semaglutide 0.005 mg/kg / DIO	Semaglutide 0.005 mg/kg / DIO	Day 21-24, 26-28	-	-	-
1B	Vehicle / DIO	Chow control	Day 4, 8, 10, 12	Day 14-15	Day 28	-
	Vehicle / DIO	NT-0796 / DIO	Day 7	Day 8	Day 9	Day 10-28
	Vehicle / DIO	Semagutide 0.001 mg/kg / DIO	Day 25, 27	-	-	-
	Vehicle / DIO	Semaglutide 0.005 mg/kg / DIO	Day 7-8	Day 9-11, 28	Day 12, 14-17	Day 13, 18-27
	Vehicle / DIO	NT-0796 + Semaglutide 0.001 mg/kg / DIO	Day 7-8	Day 9-10	Day 11, 12, 28	Day 14-27
	Vehicle / DIO	NT-0796 + Semaglutide 0.005 mg/kg / DIO	Day 6	Day 7	Day 8	Day 9-28
	NT-0796 + Semaglutide 0.005 mg/kg / DIO	NT-0796 / DIO	Day 18-22	Day 23, 24	Day 25-28	-
	NT-0796 + Semaglutide 0.005 mg/kg / DIO	Semaglutide 0.005 mg/kg / DIO	Day 14, 25	Day 15, 17, 18	Day 16, 19, 20	Day 21-24, 26-28

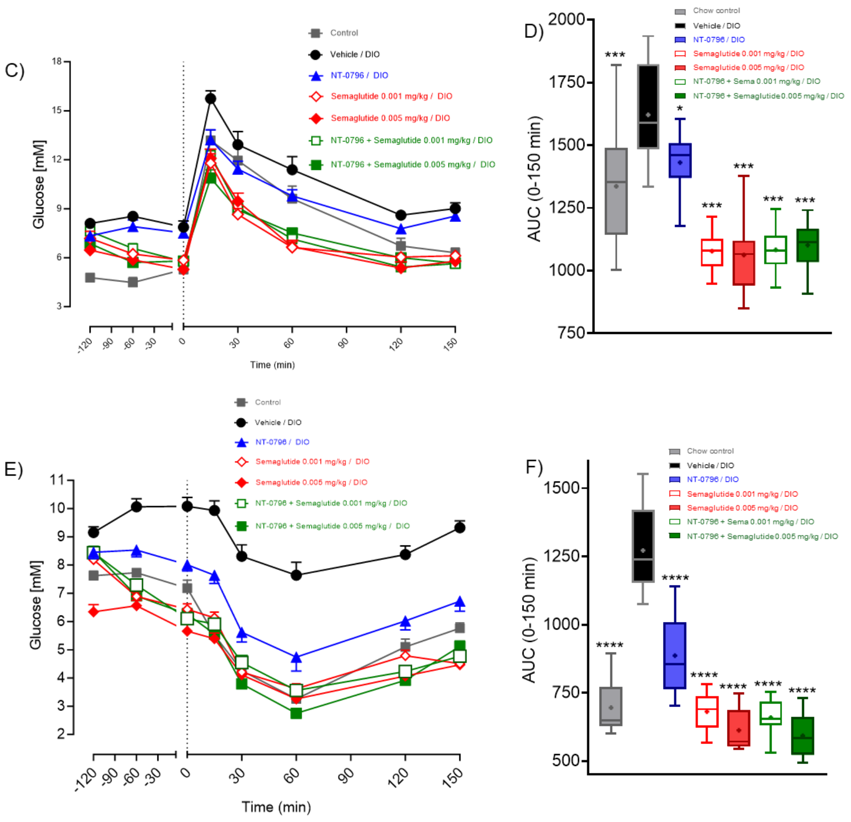

Figure S1. Effects of NT-0796 or semaglutide as monotherapy or combination in DIO mice. DIO mice fed HFD were dosed therapeutically with NT-0796 (100 mg/kg, p.o., t.i.d.), semaglutide (0.001-0.005 mg/kg, s.c., q.d.), or semaglutide (0.001-0.005 mg/kg) in

combination with NT-0796, or respective vehicle controls for 28 days. A) Experimental study design and B) statistical analysis of Figures 1A-B C) Oral glucose tolerance test (OGTT), and respective AUCs, (D). E) Insulin tolerance test (ITT), and respective AUCs, (F). Data are expressed as mean ± SEM or as box and whisker plots and analysed by one-way ANOVA with Tukey's multiple comparisons test using GraphPad Prism v10.2.2. Significance is calculated using GraphPad Prism v10.2.2, ****p<0.0001, ***p<0.001, **p<0.01, *p<0.05.

A)

Group	N	Diet	Compound Treatment Days 0-28
Chow control	9	Chow control	NA
Vehicle / DIO	10	HFD (ad libitum)	Vehicle (p.o., t.i.d.) / Vehicle (s.c., q.d.)
Semaglutide / DIO	20	HFD (ad libitum)	Vehicle (p.o., t.i.d.) / Semaglutide (0.005mpk s.c, q.d.)
NT-0796 + Sema / DIO	20	HFD (ad libitum)	NT-0796 (100mpk p.o., t.i.d.) / Semaglutide (0.005mpk s.c., q.d.)

Compound Treatment Days 29-56	N	Diet
Vehicle (p.o., t.i.d.)	10	HFD (ad libitum)
NT-0796 (100mpk, p.o., t.i.d.)	10	HFD (ad libitum)
Vehicle (p.o., t.i.d.)	10	HFD (ad libitum)
NT-0796 (100mpk, p.o., t.i.d.)	10	HFD (ad libitum)

B)

Figure	Comparison		* p<0.05	** p<0.01	*** p<0.001	**** p<0.0001
2B	Vehicle / DIO	Chow control	-	-	-	Day 0-28
	Vehicle / DIO	Semaglutide / DIO	Day 11	Day 12-15	Day 16-18	Day 19-28
	Vehicle / DIO	NT-0796 + Semaglutide / DIO	Day 8	Day 9-10	Day 11	Day 12-28
	NT-0796 + Semaglutide / DIO	Semaglutide / DIO	Day 17-20, 23-24	Day 21, 22, 25-28	-	-
	Vehicle (sema monotherapy, Day 0-28)	NT-0796 (sema monotherapy, Day 0-28)	Day 52-56	-		
	Vehicle (NT-0796 + Sema, Day 0-28)	NT-0796 (NT-0796 + Sema, Day 0-28)	Day 42-47, 49, 50	Day 48, 51-56	-	-

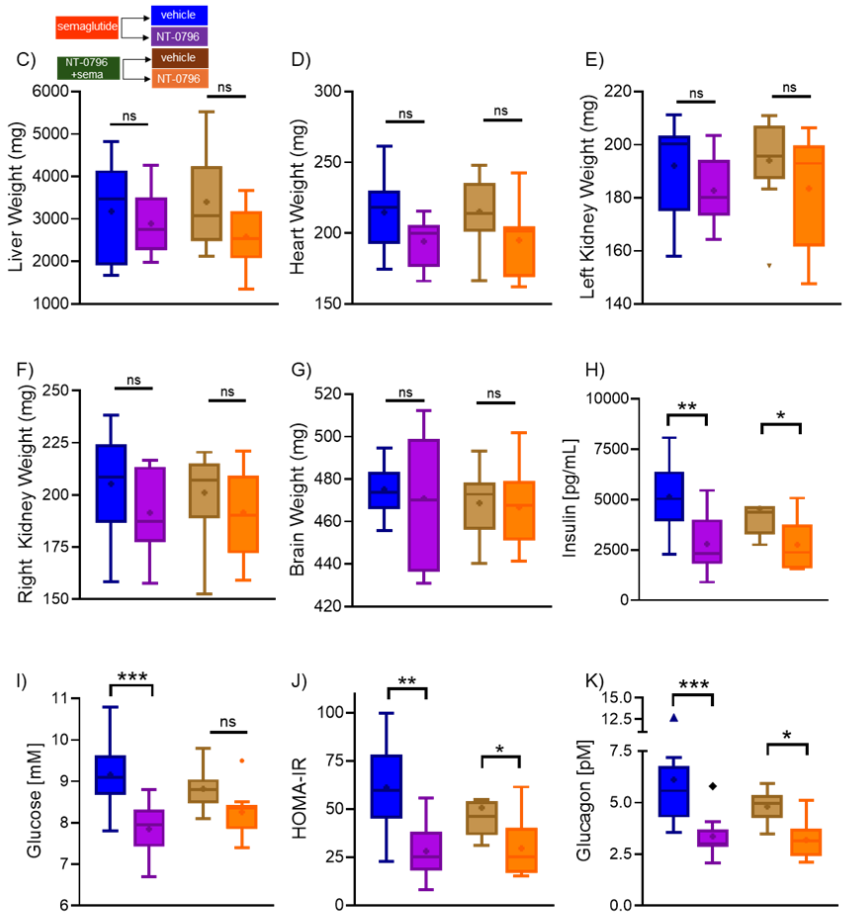

Figure S2. Effects of NT-0796 maintenance therapy on organ weights and diabetic endpoints following cessation of semaglutide in DIO mice. DIO mice were maintained on NT-0796 (100 mg/kg, p.o., t.i.d.) or vehicle treatment for 28 days, following an initial period of semaglutide or NT-0796/semaglutide therapy. A) Experimental study design and B) statistical output from Figure 2B. C) Liver,D) heart, E) left kidney, F) right kidney or G) brain weights

were assessed at study end. H) Fasting insulin, I) fasting glucose, J) HOMA-IR or K) fasting glucagon were assessed at study end. Data are expressed as box and whisker plots and analysed by one-way ANOVA with Tukey's multiple comparisons test using GraphPad Prism v10.2.2. Significance is calculated using GraphPad Prism v10.2.2, ***$p<0.001$, **$p<0.01$, *$p<0.05$.

A)

Group	N	Compound Treatment Days 0-28<br>Diet: HFD ad libitum unless stated	Compound Treatment Days 29-56<br>Diet: PUFA ad libitum unless stated
Control chow	10	normal chow only	normal chow only
DIO control	9	Vehicle (p.o., t.i.d.) / Vehicle (s.c., q.d.)	
NT-0796 / DIO	10	NT-0796 (100mpk p.o., t.i.d.) / Vehicle (s.c., q.d.)	NT-0796 (100mpk p.o., t.i.d.) /Vehicle (s.c., q.d.
Sema / DIO	9	Vehicle (p.o., t.i.d.) / Semaglutide 0.005 mpk (s.c., q.d.)	Vehicle (p.o., t.i.d.) /Semaglutide 0.005 mpk (s.c., q.d.)
NT-0796 + Sema / DIO	10	NT-0796 100mpk (p.o., t.i.d.) /Semaglutide 0.005 mpk (s.c., q.d.)	NT-0796 100mpk (p.o., t.i.d.) /Semaglutide 0.005 mpk (s.c., t.i.d.)
Calorie Restriction	9	Calorie restriction HFD	Calorie restriction PUFA

B)

Figure	Comparison		* p<0.05	** p<0.01	*** p<0.001	**** p<0.0001
4B	DIO control	Chow control	Day 14, 15, 20-22, 53-55	Day 1, 2, 6, 10-13, 16-17	Day 3, 5, 7-9	Day 4
	DIO control	NT-0796 / DIO	Day 10-12	Day 13	Day 14, 29-34	Day 15-28, 35-56
	DIO control	Semaglutide / DIO	Day 9	Day 10, 11	-	Day 12-56
	DIO control	NT-0796 + Semaglutide / DIO	-	Day 6	-	Day 7-56
	DIO control	Calorie restriction / DIO	Day 6,7	Day 8, 11	Day 10-17	Day 9, 12-16, 18-56
	NT-0796 + Semaglutide / DIO	NT-0796 / DIO	-	Day 7-10	Day 11	Day 12-56
	NT-0796 + Semaglutide / DIO	Semaglutide / DIO	Day 9-11	Day 12	Day 14	Day 13, 15-56
4C	DIO control	Control chow	-	-	Day 50-56	Day 29-49
	DIO control	NT-0796 + Semaglutide / DIO	-	Day 29-32	Day 33-40	Day 41-56

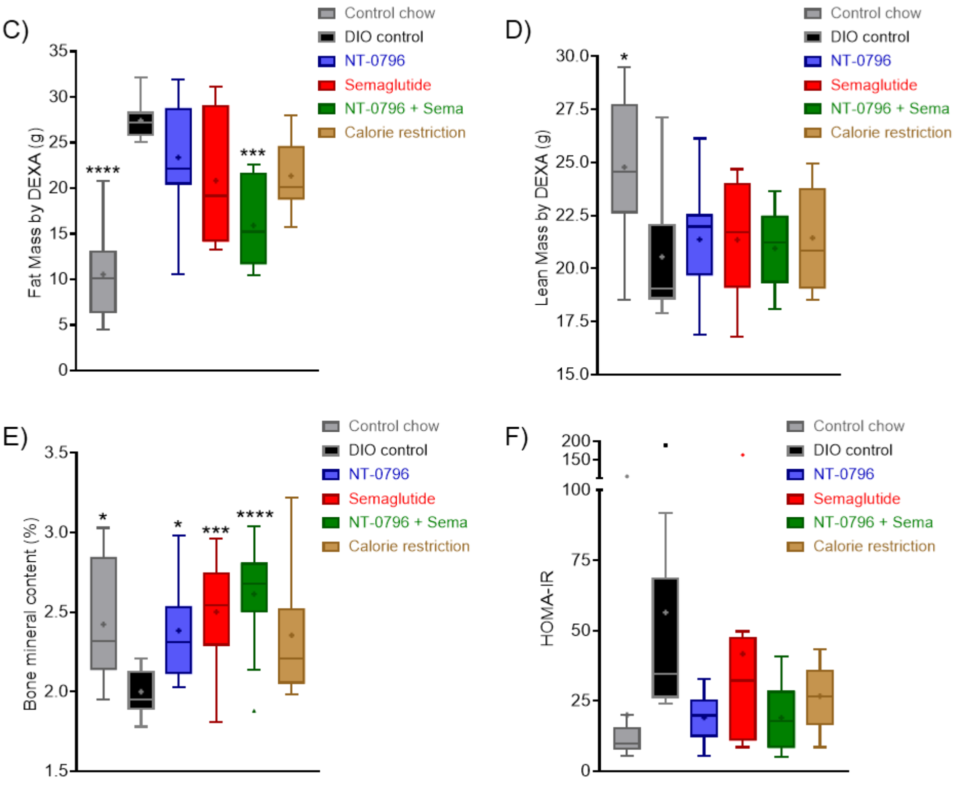

Figure S3. Effects of NT-0796, semaglutide or combinations in DIO mice switched to a PUFA diet. DIO mice fed HFD were dosed therapeutically with NT-0796 (100 mg/kg, p.o., t.i.d.), semaglutide (0.005 mg/kg, s.c., q.d.), or in combination from day 0-28. On day 29, diet was switched to PUFA diet and the respective treatment continued (over days 29-56). An

additional group of mice served as calorie restricted controls whose body weights were maintained as close to NT-0796-dosed mice as possible by controlling the degree of calorie restriction throughout the experiment. A) Experimental study design. B) Statistical output from Figure 4B-C. C) Fat mass , D) lean mass and E) bone mineral content were evaluated by body composition DEXA scans at day 28. F) HOMA-IR was assessed at day 55. Data are expressed as box and whisker plots and analysed by one-way ANOVA with Tukey's multiple comparisons test using GraphPad Prism v10.2.2. Significance is calculated using GraphPad Prism v10.2.2, ****p<0.0001, ***p<0.001, **p<0.01

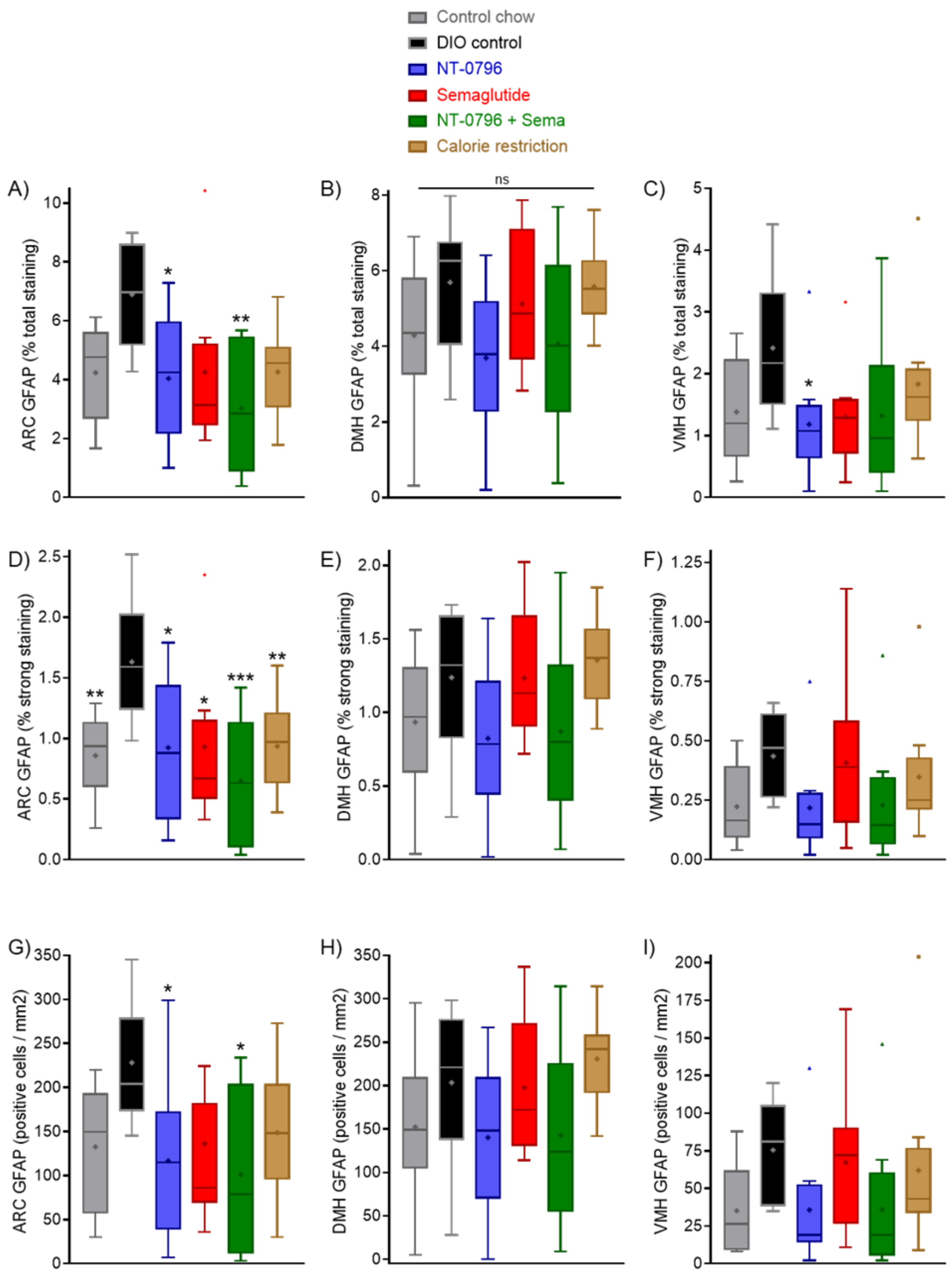

Figure S4. Effects of NT-0796, semaglutide or combinations on hypothalamic GFAP. GFAP immunoreactivity was assessed within the arcuate nucleus (ARC) and dorsomedial hypothalamus (DMH) and ventral medial hypothalamus (VMH) of DIO mice following continuous dosing with NT-0796 (100 mg/kg, p.o., t.i.d.), semaglutide (0.005 mg/kg, s.c., q.d.), their combinations, or calorie restriction over 56 days in DIO mice. Total GFAP staining (%) in the (A) ARC, (B) DMH or (C) VMH. Percentage strong GFAP staining in the (D) ARC, (E) DMH or (F) VMH. Total GFAP positive cell numbers per mm^2, within the (G) ARC, (H) DMH or (I) VMH was assessed. Data are expressed as box and whisker plots and analysed by one-way

ANOVA with Tukey's multiple comparisons test using GraphPad Prism v10.2.2. Significance is calculated using GraphPad Prism v10.2.2, **$p<0.01$, *$p<0.05$.

Supplementary Methods

Details of the diet-induced obesity models

All *in vivo* experimental procedures were approved by the Institutional Animal Care and Use Committee (IACUC) at HDB. Animal husbandry including acclimatization period, housing and conditions, including lighting (12 h light / 12 h dark; 8:00 am the beginning of lights on cycle) have been previously described (18).NT-0796 was prepared at 10 mg/mL in a vehicle of 0.5% methylcellulose (Sigma, Cat#M7140) and 0.2% Tween 80 (Sigma, Cat#P4780) with a dosing volume of 10 mL/kg (p.o., t.i.d.). Semaglutide (Aladdin, Cat#S304954) was prepared at a final concentration of 0.005 mg/mL and 0.001 mg/mL in a vehicle of 0.05% Tween 80 in PBS (Gibco, Cat#02100) with a dosing volume of 10 mL/kg (s.c.; q.d.). Oral gavages (t.i.d.) were performed at 08:00 am, 03:00 pm, and 10:00 pm. Subcutaneous injections were given once daily at 08:30 am. T.i.d. oral gavage dosing of NT-0796 was conducted to compensate for its short half-life in mouse, and to ensure ≥50% predicted brain target cover (for the active metabolite, NDT-19795) throughout the study, as previously described (18). Percentage of fat and lean tissue were measured using InAlyzer DEXA system (InAlyzer DXA, Medikors Inc., Seoul, Korea). At study end, animals were sacrificed using CO2 inhalation and all efforts were made to minimize suffering. Organ (liver, heart, brain and kidneys) and adipose tissues (perirenal, inguinal and epididymal) were collected and weighed. Protocol information specific to each figure is detailed below.

Figure 1*: Effects of the NLRP3 inhibitor, NT-0796, on efficacy of low dose semaglutide in a mouse model of DIO.*

All hCES-1 mice were fed with normal chow until 6 weeks of age, then the diet was switched to HFD for additional 15 weeks before the study (except mice on normal chow, Figure S1A). Animals were randomized to groups based on body weight and plasma HDL, LDL, TC and TG, then compound dosing commenced. Respective vehicles were administered to controls as detailed in Figure S1A. Food intake was measured daily. All animals were group housed, and food intake was measured by group (cage). At 8:00 am every day (the beginning of light cycle; lights on), food pellet was weighed, and was provided to the animals in the cage *ad libitum*. At 8:00 am on the following day (the beginning of light cycle; lights on), the remaining food pellet was weighed again, and the difference was recorded as the total food consumption by the group of animals in 24 hours. The final results were expressed as average food consumption per mice (g; total food consumption/number of animals in the cage). Body weight was measured daily after food intake measurement. At the end of the study on day 28, blood samples were collected via cardiac puncture into tubes pre-coated with EDTA-K2 for plasma preparation. Plasma samples were obtained by centrifugation at 4000 rpm for 10 minutes at 4°C and then stored at -80°C prior to biomarker assessment. Overnight fasting was performed into day 25 prior to an OGTT test as described below. A 4-hour fast on day 27 was performed prior to an ITT tests conducted as described below.

Figure 2-3*: Effects of the NLRP3 inhibitor, NT-0796, on efficacy of low dose semaglutide in a mouse model of DIO.*

All hCES-1 mice were fed with normal chow until 5 weeks of age, then was switched to HFD for an additional 15 weeks (except for chow fed mice). Animals were randomized, group housed and food intake / body weight assessments measured as for the above study (in Figure 1). During days 0-28, mice were dosed with NT-0796, semaglutide or a combination as detailed in Figure S2A. At day 28, the semaglutide monotherapy, or NT-0796-semaglutide combination dosed DIO mice were maintained on HFD for a further 28 days (days 29-56). However, during days 29-56, their dosing regimes were switched to NT-0796 monotherapy or

respective vehicle only. Body composition analysis (DEXA) was performed on days 0, 28 and 56. On day 55, mice were fasted for 4 hours and blood glucose assessed from a 3 µL blood sample using the tail nick method. At the end of the study on day 56, blood samples were collected via cardiac puncture into tubes pre-coated with EDTA-K2 for plasma preparation. Plasma samples were obtained by centrifugation at 4000 rpm for 10 minutes at 4°C and then stored at -80°C prior to biomarker analysis. At the end of the study, kidney, brain, liver and heart organ were immediately collected and organ weights recorded. Perirenal, inguinal and epididymal adipose tissue were collected and weights were recorded.

Figure 4-5*: Effects of the NLRP3 inhibitor, NT-0796, on efficacy of low dose semaglutide in a mouse model of DIO.*

All hCES-1 mice were fed with normal chow until 6 weeks of age, then was switched to HFD for an additional 15 weeks (except for chow fed mice). As detailed in Figure S3A, the whole study consisted of two parts. In part one (days 0-28), the DIO animals continued to receive HFD diet whilst being dosed with compounds. In part two (days 29-56), compound dosing continued, but all DIO mice were switched from the HFD to a PUFA-enriched diet. Compound dosing consisted of NT-0796, semaglutide or a combination as detailed in Figure S3A. A calorie restricted group of mice was included throughout the study, whose body weights were matched as closely as possible to that of mice receiving NT-0796 monotherapy by daily adjustment of the allocated food (HFD for days 0-28, or PUFA for days 29-56). This allowed the effects of NT-0796 to be assessed, as opposed to effects secondary to weight loss. Chow fed control animals remained on normal chow throughout the 56 days. Animals were randomized, group housed with food intake / body weight assessments, DEXA and blood glucose measured as for the prior studies. Overnight fasting was performed into day 55 to enable the analysis of further exploratory endpoints. Terminal blood for plasma, organs and adipose tissues were collected and weighed at study end as described for study in Figure 2, above.

Further statistical information

For box and whisker plots, the box extends from the 25th to 75th percentiles and within the box, the line is the median, "+" is the mean. The whiskers extend to extreme values within 1.5x the interquartile range and outliers beyond this are shown as individual points.

Food intake and body weight measurement

Food intake, body weight and percentage body weight change were assessed daily (18). Average daily calorie intake was calculated from food intake and calorie content of each diet on a per gram basis.

Drug exposure analysis

Sample collection, stabilization, preparation and LC/MS/MS analysis for the quantification of NT-0796 and NDT-19795 exposure were performed as previously described (18). For semaglutide pharmacokinetics analysis, naïve hCES1 mice were dosed to steady state with semaglutide (0.005 mg/kg; s.c., q.d.) alone, or in combination with NT-0796 (100 mg/kg, p.o., t.i.d.). Plasma was collected 0.75, 2.5 and 4.5 hours after their final morning dose and semaglutide levels analysed by ELISA (Creative-diagnostics, Cat#DEIASL092).

Plasma biomarker analysis

ELISAs were used to measure plasma levels of NLRP3-dependent inflammation markers sVCAM-1 (R&D, Minneapolis, USA, Cat#MVC00) and PCSK9 (Sino Biological, Beijing, China, Cat#KIT50251). An Meso Scale Discovery (MSD) U-PLEX® assay was used to measure acute phase protein, IL-1RA. IL-1RA, sVCAM-1, PCSK9 are downstream markers of NLRP3-dependent cytokine IL-1, and have previously been used to verify NT-0796 target engagement

in DIO mice (18). Levels of leptin (R&D, McKinley Place NE, USA, Cat#MOB00B), adiponectin (R&D, McKinley Place NE, USA, Cat#MRP300), suPAR (R&D, McKinley Place NE, USA, Cat#DY531), were measured by ELISA. Levels of plasma TC, TG, LDL and HDL were measured with kits from FUJIFILM (FUJIFILM Wako Pure Chemical Corporation, Osaka, Japan). Levels of glucagon and insulin were measured using MSD Single-PLEX® assays (Cat#K150U5D-2 and Cat#K152BZC, respectively). HOMA-IR was calculated for each animal using the following equation = fasting serum insulin (mU/l) × fasting serum glucose (mmol/l))/22.5.

GFAP immunohistochemistry

Coronal brain sections (bregma, AP −1.46 mm to -1.82) were collected for immunohistochemical assessment of hypothalamic glial fibrillary acid protein (GFAP) expression as previously described (18). Briefly, following assignment of the arcuate nucleus (ARC), dorsomedial hypothalamic nucleus (DMH) and ventromedial hypothalamic nucleus (VMH) areas, the percentage of areas displaying weak, moderate and strong GFAP immunoreactivity were quantified using optical density (OD) threshold values of 0.15, 0.25 and 0.35, respectively in the Multiplex IHC v3.2.3 module of the HALO® Image Analysis Platform (Indica Labs, Albuquerque, NM, USA). The average total (weak, moderate or strong) GFAP staining (%), average strong GFAP staining (%), or the total number of GFAP positive cells (per mm^2) across the ARC, DMH and VMH were calculated and compared across groups.

IL-1α Pulldown and Western Blot Detection

Mouse liver homogenates were prepared in homogenisation buffer containing 1% TritonX-100 (Sigma-Aldrich, Cat#TX-100) supplemented with one Pierce Protease Inhibitor Mini Tablet, ethylenediaminetetraacetic acid (EDTA)-free (Thermo Fisher, Cat#A32955) per 10 mL buffer, 1 mM final concentration EDTA (Sigma-Aldrich, Cat#324503) and 1 mM final concentration of

iodoacetamide (Sigma-Aldrich Cat#I1149) in PBS. Homogenates were prepared in 2 mL MP Lysing Matrix D bead tubes (MP Biomedicals, LLC, USA, Cat#MP 6913100), using 1 mL of homogenisation buffer per sample using an MP FastPrep 24 bead homogeniser (MP Biomedicals, LLC, USA, Cat#116004500). Samples were homogenised 2 or 3 times for 40-second cycles at 6.5 m/s. After homogenization, samples were centrifuged for 30 minutes, 17000 × *g* at 4°C to remove insoluble material and supernatant was transferred to a fresh tube then either stored at -80°C or analysed further. Protein concentrations were determined using Pierce BCA Protein Assay Kits (Thermo Fisher, Cat#23227). 2 µg of mouse IL-1R1-Fc capture protein (R&D, Cat#771-MR-100) in PBS was added to each sample, mixed and incubated on ice for 2 hours. During the incubation, protein A beads were washed three times with PBS-1% TX-100 with inhibitors (Pierce Protein A Plus Agarose, Thermo Fisher, Cat#22812]). Final beads were suspended to produce a 20% slurry. At the end of the 2-hour incubation, 50 µL of the protein A bead suspension was added to each sample (10 µL bead volume) and incubated for 1 hour at 4°C with continuous mixing. Beads were washed 3× with 500 µL PBS-1% TX-100 with inhibitors and the washed beads were stored at -80 until analysis. For total extracts, 50 µg total protein was loaded per lane. Samples were diluted with an equal volume of 2x reducing sample buffer, heated to 95°C for 5 minutes, and centrifuged for 1 minute at 17000 × *g* prior to loading onto gel. For the pulldowns, 40 µL of 1× reducing sample buffer was added to the washed beads, and the samples were heated to 95°C for 5 minutes, centrifuged for 1 minute at 17000 × *g*, and loaded. 10 pg of recombinant mouse IL-1alpha (R&D Systems, Cat#400-ML) diluted in sterile PBS-0.1% BSA was run in an adjacent lane as a positive control for western detection. Thermo Fisher Bolt 4-12% gels were used for electrophoresis with running buffer of Bolt MES SDS Running Buffer (Thermo Fisher, Cat#B0002). Transfer was conducted using a nitrocellulose on a PowerBlotter device, using Power Blotter Select Transfer Stacks (Thermo Fisher, Cat#PB3310) set on a standard Mixed MW program for transfer. For detection, blocking was performed in a 1:1 mix of Licor Intercept (Licor, Cat#927-70001) block and SuperBlock (Thermo Fisher, Cat#37515) for 1 hour at room temperature (RT) with mixing. Samples were incubated with primary IL-1α antibodies (goat anti-mouse IL-

1α (R&D, #AF- 400-NA) 0.1 mg/mL in Intercept:SuperBlock containing-0.1% Tween-20 overnight at 4°C with continuous mixing. Following blocking, samples were washed three times for 10 minutes with PBS-0.1% Tween-20. Samples were then incubated with detection antibodies (was donkey anti-goat (H+L) AlexaFluor Plus 800 highly cross adsorbed; Thermo Fisher, Cat#A32930; 0.1 µg/mL in Intercept: Superblock containing 0.1% Tween-20) in the dark at room temperature with continuous mixing. Samples were then washed six times with PBS-0.1% Tween 20 then scanned on a Licor Odyssey (LICORBio) using an 800 nm channel.

Oral glucose tolerance test

Animals were fasted overnight. The following day animals were weighed and dosing volume for 20% glucose solution (p.o.) was calculated (final concentration of 2 g glucose/kg and a maximum dosing volume of 10 mL/kg body weight). Blood glucose measurements were performed using ACCU-CHEK Active Blood Glucose Meter (Roche, Cat#06993788001), with ACCU-CHEK Active Test Strips (Roche, Cat#REF03146662) following manufacturer's instructions. Approximately 3 µL of blood/animal/test strip was added by making a small incision (tail nick) over the lateral tail vein (1 to 2 cm from the tail top) using a scalpel blade followed by gentle tapping of test strip against the incision. Bleeding was subsequently stopped by placing direct pressure to the incision site until the formation of blood clots after which animals were returned to their cages. At the 0-minute time-point, blood glucose level measurements were repeated by starting with the animal first gavaged and keeping to the same time intervals between animals until the entire cohort had been measured. Bleeding was restarted by gently removing the clot from the first incision site. Glucose solution (Sigma, Cat#G8270) was administered by oral gavage to each animal with a 30- to 60-second interval between animals. Glucose test was repeated using the above described method at 15, 30, 60, 120 and 150 minute following first gavage.

Insulin tolerance test

Animals were fasted for 4 hours for Insulin tolerance test (ITT), weighed and dosed with 0.5 IU/kg insulin (i.p.; Sanofi, Cat#SJ20200023). Blood glucose measurements were performed as described in the OGTT methods.
